# Lithium difluoro­(oxalato)borate tetra­methyl­ene sulfone disolvate

**DOI:** 10.1107/S1600536811011743

**Published:** 2011-04-07

**Authors:** Joshua L. Allen, Paul D. Boyle, Wesley A. Henderson

**Affiliations:** a Ionic Liquids & Electrolytes for Energy Technologies (ILEET) Laboratory, Department of Chemical and Biomolecular Engineering, North Carolina State University, 911 Partners Way, Raleigh, NC 27695, USA; b X-ray Structural Facility, Department of Chemistry, North Carolina State University, 2620 Yarbrough Drive, Raleigh, NC 27695, USA

## Abstract

The title compound, Li^+^·C_2_BF_2_O_4_^−^·2C_4_H_8_O_2_S, is a dimeric species, which resides across a crystallographic inversion center. The dimers form eight-membered rings containing two Li^+^ cations, which are joined by O_2_S sulfone linkages. The Li^+^ cations are ligated by four O atoms from the anions and solvent mol­ecules, forming a pseudo-tetra­hedral geometry. The exocyclic coordination sites are occupied by O atoms from the oxalate group of the difluoro­(oxalato)borate anion and an additional tetra­methyl­ene sulfone ligand.

## Related literature

For physiochemical properties of tetra­methyl­ene sulfone (TMS), see: Della Monica *et al.* (1968 ▶); Dudley *et al.* (1991 ▶); Domanska *et al.* (1996 ▶). For electrochemical properties of TMS, see: Xu & Angell (2002 ▶); Abouimrane *et al.* (2009 ▶); Sun & Angell (2009 ▶). For electrochemical properties of lithium difluoro­(oxalato)borate (LiDFOB), see: Zhang (2007 ▶); Chen *et al.* (2007 ▶); Fu *et al.* (2010 ▶). 
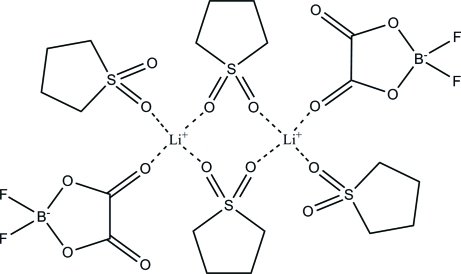


## Experimental

### 

#### Crystal data

Li^+^·C_2_BF_2_O_4_^−^·2C_4_H_8_O_2_S
*M* *_r_* = 384.12
Monoclinic, 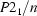

*a* = 13.9005 (4) Å
*b* = 5.8917 (1) Å
*c* = 19.9627 (5) Å
β = 106.0101 (13)°
*V* = 1571.48 (7) Å^3^
*Z* = 4
Mo *K*α radiation
μ = 0.40 mm^−1^
*T* = 110 K
0.51 × 0.17 × 0.16 mm

#### Data collection

Bruker–Nonius Kappa X8 APEXII diffractometer
Absorption correction: multi-scan (*SADABS*; Bruker, 2009 ▶) *T*_min_ = 0.823, *T*_max_ = 0.939
59573 measured reflections
6723 independent reflections
4514 reflections with *I* > 2σ(*I*)
*R*_int_ = 0.054

#### Refinement

*R*[*F* ^2^ > 2σ(*F* ^2^)] = 0.044
*wR*(*F* ^2^) = 0.113
*S* = 1.02
6723 reflections
281 parameters
All H-atom parameters refined
Δρ_max_ = 0.80 e Å^−3^
Δρ_min_ = −0.45 e Å^−3^

### 

Data collection: *APEX2* (Bruker, 2009 ▶); cell refinement: *SAINT* (Bruker, 2009 ▶); data reduction: *SAINT*; program(s) used to solve structure: *SIR92* (Altomare *et al.*, 1994 ▶); program(s) used to refine structure: *SHELXTL* (Sheldrick, 2008 ▶); molecular graphics: *ORTEP-3* (Farrugia, 1997 ▶); software used to prepare material for publication: *cif2tables.py* (Boyle, 2008 ▶).

## Supplementary Material

Crystal structure: contains datablocks I, global. DOI: 10.1107/S1600536811011743/si2348sup1.cif

Structure factors: contains datablocks I. DOI: 10.1107/S1600536811011743/si2348Isup2.hkl

Additional supplementary materials:  crystallographic information; 3D view; checkCIF report

## Figures and Tables

**Fig. 1.**
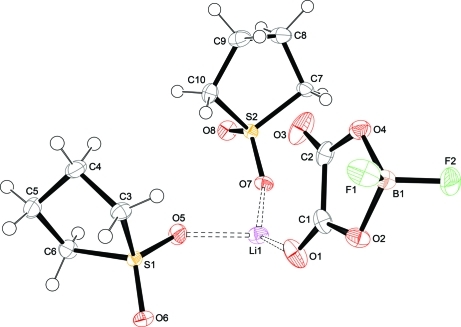
Asymmetric unit of (TMS)2:LiDFOB. Thermal ellipsoids are at 50% probability (Li-purple, O-red, F-green, B-tan, C-grey, S-yellow).

**Fig. 2.**
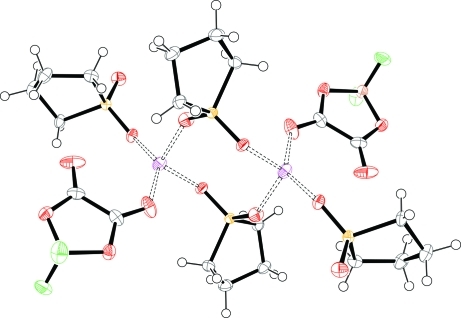
Ion and solvent coordination in (TMS)2:LiDFOB. Thermal ellipsoids are at 50% probability (Li-purple, O-red, F-green, B-tan, C-grey, S-yellow).

**Fig. 3.**
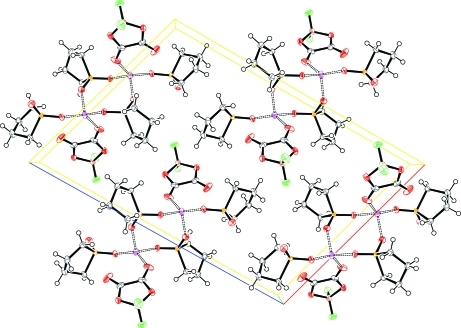
Unit cell of (TMS)2:LiDFOB. Thermal ellipsoids are at 50% probability (Li-purple, O-red, F-green, B-tan, C-grey, S-yellow).

## supplementary crystallographic information

### Comment

Solvate crystal structures provide invaluable information for understanding the ionic association tendency and manner in which anions and solvent molecules coordinate Li^+^ cations. Understanding the solid-state behavior provides insight into the various solvates that may exist in liquid solvent-lithium salt electrolytes utilized in state-of-the-art Li-ion batteries. The physiochemical and electrochemical properties of both tetramethylene sulfone (TMS) and lithium difluoro(oxalato)borate (LiDFOB) have attracted much attention recently for non-aqueous secondary battery applications.

The Li^+^ cation in the title structure, which resides across a crystallographic inversion center, is coordinated by two sulfonyl O atoms from TMS and a carbonyl O atom from the DFOB^-^ anion (Fig. 1). An eight member dimer ring structure is formed from this coordination by linking two Li^+^ cations through their coordination by TMS molecules coordinated to both Li^+^ cations with each cation coordinated by a different sulfonyl oxygen (Fig. 2). The eight membered rings are packed in the crystal structure in layers such that *Z* = 2 (Fig. 3).

### Experimental

LiDFOB was synthesized by the direct reaction of excess boron trifluoride diethyl etherate (BF_3_-ether) with lithium oxalate (oxalic acid dilithium salt), both used as-received from Sigma-Aldrich, and extracted/recrystallized from dimethyl carbonate (DMC). The DMC-LiDFOB solvate was vacuum dried at 378 K for 48 h, yielding a high purity salt. TMS (Sigma-Aldrich, >99.8%) was used as-received. A solution was made by dissolving LiDFOB (1.566 mmol) in TMS (6.264 mmol) at 353 K. The solution was allowed to slowly cool to room temperature. Colorless crystals formed suitable for X-ray analysis on standing.

### Refinement

The structure was solved by direct methods using the *SIR92* program. All non-hydrogen atoms were obtained from the initial solution. The hydrogen atoms were introduced at idealized positions and were allowed to refine isotropically. The structural model was fit to the data using full matrix least-squares based on *F*^2^. The calculated structure factors included corrections for anomalous dispersion from the usual tabulation. The structure was refined using the XL program from *SHELXTL* (Sheldrick, 2008). Graphic plots were produced using the *ORTEP-3* program.

### Crystal data

**Table:**

Li^+^·C_2_BF_2_O_4_^−^·2C_4_H_8_O_2_S	*F*(000) = 792
*M**_r_* = 384.12	*D*_x_ = 1.623 Mg m^−^^3^
Monoclinic, *P*2_1_/*n*	Melting point: 367.85 K
Hall symbol: -P 2yn	Mo *K*α radiation, λ = 0.71073 Å
*a* = 13.9005 (4) Å	Cell parameters from 9927 reflections
*b* = 5.8917 (1) Å	θ = 3.1–31.8°
*c* = 19.9627 (5) Å	µ = 0.40 mm^−^^1^
β = 106.0101 (13)°	*T* = 110 K
*V* = 1571.48 (7) Å^3^	Prism, colourless
*Z* = 4	0.51 × 0.17 × 0.16 mm

### Data collection

**Table:**

Bruker–Nonius Kappa X8 APEXII diffractometer	6723 independent reflections
Radiation source: fine-focus sealed tube	4514 reflections with *I* > 2σ(*I*)
graphite	*R*_int_ = 0.054
ω and phi scans	θ_max_ = 35.0°, θ_min_ = 2.1°
Absorption correction: multi-scan (*SADABS*; Bruker, 2009)	*h* = −22→22
*T*_min_ = 0.823, *T*_max_ = 0.939	*k* = −9→8
59573 measured reflections	*l* = −31→31

### Refinement

**Table:**

Refinement on *F*^2^	Primary atom site location: structure-invariant direct methods
Least-squares matrix: full	Secondary atom site location: difference Fourier map
*R*[*F*^2^ > 2σ(*F*^2^)] = 0.044	Hydrogen site location: inferred from neighbouring sites
*wR*(*F*^2^) = 0.113	All H-atom parameters refined
*S* = 1.02	*w* = 1/[σ^2^(*F*_o_^2^) + (0.0531*P*)^2^ + 0.5209*P*] where *P* = (*F*_o_^2^ + 2*F*_c_^2^)/3
6723 reflections	(Δ/σ)_max_ = 0.001
281 parameters	Δρ_max_ = 0.80 e Å^−^^3^
0 restraints	Δρ_min_ = −0.45 e Å^−^^3^

### Special details

**Table:**

Geometry. All e.s.d.'s (except the e.s.d. in the dihedral angle between two l.s. planes) are estimated using the full covariance matrix. The cell e.s.d.'s are taken into account individually in the estimation of e.s.d.'s in distances, angles and torsion angles; correlations between e.s.d.'s in cell parameters are only used when they are defined by crystal symmetry. An approximate (isotropic) treatment of cell e.s.d.'s is used for estimating e.s.d.'s involving l.s. planes.
Refinement. Refinement of *F*^2^ against ALL reflections. The weighted *R*-factor *wR* and goodness of fit *S* are based on *F*^2^, conventional *R*-factors *R* are based on *F*, with *F* set to zero for negative *F*^2^. The threshold expression of *F*^2^ > σ(*F*^2^) is used only for calculating *R*-factors(gt) *etc*. and is not relevant to the choice of reflections for refinement. *R*-factors based on *F*^2^ are statistically about twice as large as those based on *F*, and *R*- factors based on ALL data will be even larger.

### Fractional atomic coordinates and isotropic or equivalent isotropic displacement parameters (Å^2^)

**Table:**

	*x*	*y*	*z*	*U*_iso_*/*U*_eq_	
Li1	0.3460 (2)	−0.0347 (4)	0.00559 (15)	0.0214 (5)	
O1	0.31626 (9)	0.20781 (19)	−0.06478 (8)	0.0316 (3)	
O2	0.22276 (8)	0.47559 (18)	−0.13303 (6)	0.0222 (2)	
O3	0.16743 (13)	0.2577 (2)	0.01240 (7)	0.0398 (4)	
O4	0.10533 (8)	0.52447 (19)	−0.06874 (6)	0.0215 (2)	
C1	0.24614 (12)	0.3388 (2)	−0.07979 (9)	0.0216 (3)	
C2	0.16898 (13)	0.3664 (3)	−0.03845 (8)	0.0225 (3)	
B1	0.13278 (13)	0.6151 (3)	−0.13039 (9)	0.0197 (3)	
F1	0.15911 (8)	0.84008 (16)	−0.12160 (6)	0.0318 (2)	
F2	0.05644 (8)	0.58017 (19)	−0.18987 (5)	0.0320 (2)	
S1	0.53657 (3)	0.23623 (5)	0.095635 (17)	0.01352 (8)	
O5	0.45522 (9)	0.07304 (19)	0.08378 (6)	0.0241 (2)	
O6	0.58816 (8)	0.25394 (17)	0.04173 (5)	0.0185 (2)	
C3	0.49317 (12)	0.5049 (2)	0.11463 (8)	0.0192 (3)	
H3A	0.5342 (17)	0.620 (4)	0.1003 (11)	0.037 (6)*	
H3B	0.4301 (16)	0.517 (3)	0.0880 (11)	0.029 (5)*	
C4	0.50818 (13)	0.4926 (3)	0.19321 (8)	0.0234 (3)	
H4A	0.4597 (15)	0.390 (3)	0.2058 (10)	0.025 (5)*	
H4B	0.4995 (18)	0.640 (4)	0.2119 (13)	0.048 (7)*	
C5	0.61283 (13)	0.3906 (3)	0.22363 (9)	0.0253 (3)	
H5A	0.6587 (17)	0.502 (4)	0.2182 (12)	0.038 (6)*	
H5B	0.6283 (16)	0.349 (4)	0.2732 (11)	0.029 (5)*	
C6	0.62066 (13)	0.1822 (3)	0.17993 (8)	0.0204 (3)	
H6A	0.5960 (16)	0.054 (4)	0.1967 (11)	0.033 (6)*	
H6B	0.6831 (15)	0.159 (3)	0.1732 (10)	0.020 (5)*	
S2	0.22934 (3)	−0.23755 (5)	0.111283 (18)	0.01444 (8)	
O7	0.27513 (8)	−0.22574 (17)	0.05399 (6)	0.0193 (2)	
O8	0.25996 (9)	−0.42689 (18)	0.15829 (6)	0.0234 (2)	
C7	0.09674 (12)	−0.2291 (3)	0.08044 (9)	0.0203 (3)	
H7A	0.0718 (15)	−0.376 (4)	0.0698 (11)	0.031 (5)*	
H7B	0.0845 (16)	−0.138 (4)	0.0414 (12)	0.037 (6)*	
C8	0.06790 (13)	−0.1165 (3)	0.14076 (10)	0.0263 (3)	
H8A	−0.0049 (15)	−0.066 (3)	0.1276 (10)	0.024 (5)*	
H8B	0.0803 (18)	−0.232 (4)	0.1822 (13)	0.041 (6)*	
C9	0.13774 (13)	0.0881 (3)	0.16254 (10)	0.0251 (3)	
H9A	0.1127 (17)	0.211 (4)	0.1286 (12)	0.032 (6)*	
H9B	0.1384 (16)	0.142 (4)	0.2075 (12)	0.034 (6)*	
C10	0.24416 (12)	0.0200 (2)	0.16109 (8)	0.0197 (3)	
H10A	0.2745 (14)	0.127 (3)	0.1391 (10)	0.022 (5)*	
H10B	0.2877 (16)	−0.014 (4)	0.2038 (12)	0.032 (5)*	

### Atomic displacement parameters (Å^2^)

**Table:**

	*U*^11^	*U*^22^	*U*^33^	*U*^12^	*U*^13^	*U*^23^
Li1	0.0245 (13)	0.0185 (11)	0.0241 (13)	−0.0036 (10)	0.0115 (11)	−0.0014 (10)
O1	0.0206 (6)	0.0172 (5)	0.0517 (8)	0.0016 (4)	0.0012 (6)	0.0037 (5)
O2	0.0217 (5)	0.0197 (5)	0.0279 (6)	0.0022 (4)	0.0115 (5)	0.0050 (4)
O3	0.0674 (10)	0.0299 (7)	0.0198 (6)	−0.0123 (6)	0.0080 (6)	0.0062 (5)
O4	0.0223 (5)	0.0235 (5)	0.0206 (5)	−0.0022 (4)	0.0092 (4)	−0.0007 (4)
C1	0.0183 (7)	0.0144 (6)	0.0283 (8)	−0.0032 (5)	0.0004 (6)	0.0010 (5)
C2	0.0298 (8)	0.0184 (6)	0.0165 (7)	−0.0073 (6)	0.0016 (6)	0.0005 (5)
B1	0.0193 (8)	0.0188 (7)	0.0224 (8)	0.0026 (6)	0.0082 (6)	0.0040 (6)
F1	0.0331 (6)	0.0168 (4)	0.0491 (7)	0.0030 (4)	0.0175 (5)	0.0069 (4)
F2	0.0305 (6)	0.0384 (6)	0.0221 (5)	0.0087 (4)	−0.0011 (4)	0.0059 (4)
S1	0.01483 (16)	0.01248 (13)	0.01344 (14)	−0.00014 (11)	0.00423 (11)	−0.00114 (11)
O5	0.0250 (6)	0.0234 (5)	0.0240 (6)	−0.0105 (4)	0.0068 (5)	−0.0052 (4)
O6	0.0216 (5)	0.0192 (5)	0.0169 (5)	0.0028 (4)	0.0091 (4)	−0.0002 (4)
C3	0.0222 (7)	0.0164 (6)	0.0201 (7)	0.0047 (5)	0.0074 (6)	−0.0004 (5)
C4	0.0276 (8)	0.0245 (7)	0.0208 (7)	−0.0014 (6)	0.0112 (6)	−0.0066 (6)
C5	0.0260 (8)	0.0327 (8)	0.0166 (7)	−0.0059 (7)	0.0050 (6)	−0.0040 (6)
C6	0.0199 (7)	0.0229 (7)	0.0179 (7)	0.0026 (6)	0.0042 (6)	0.0060 (5)
S2	0.01413 (16)	0.01212 (14)	0.01752 (15)	−0.00119 (11)	0.00510 (12)	0.00011 (11)
O7	0.0180 (5)	0.0190 (5)	0.0235 (5)	−0.0017 (4)	0.0100 (4)	0.0003 (4)
O8	0.0293 (6)	0.0155 (5)	0.0251 (6)	0.0019 (4)	0.0068 (5)	0.0050 (4)
C7	0.0147 (6)	0.0226 (7)	0.0252 (7)	−0.0035 (5)	0.0081 (6)	−0.0056 (6)
C8	0.0258 (9)	0.0243 (7)	0.0338 (9)	−0.0026 (6)	0.0168 (7)	−0.0060 (6)
C9	0.0302 (9)	0.0205 (7)	0.0291 (8)	−0.0026 (6)	0.0158 (7)	−0.0067 (6)
C10	0.0234 (7)	0.0148 (6)	0.0192 (7)	−0.0041 (5)	0.0031 (6)	−0.0018 (5)

### Geometric parameters (Å, °)

**Table:**

Li1—O7	1.922 (3)	C4—H4A	0.99 (2)
Li1—O5	1.959 (3)	C4—H4B	0.97 (3)
Li1—O1	1.966 (3)	C5—C6	1.527 (2)
Li1—O6^i^	1.969 (3)	C5—H5A	0.94 (2)
O1—C1	1.2144 (19)	C5—H5B	0.98 (2)
O2—C1	1.3014 (19)	C6—H6A	0.93 (2)
O2—B1	1.510 (2)	C6—H6B	0.925 (19)
O3—C2	1.2053 (19)	S2—O8	1.4443 (11)
O4—C2	1.312 (2)	S2—O7	1.4559 (11)
O4—B1	1.485 (2)	S2—C7	1.7758 (16)
C1—C2	1.532 (2)	S2—C10	1.7945 (15)
B1—F2	1.372 (2)	C7—C8	1.522 (2)
B1—F1	1.373 (2)	C7—H7A	0.94 (2)
S1—O6	1.4521 (11)	C7—H7B	0.92 (2)
S1—O5	1.4530 (11)	C8—C9	1.534 (2)
S1—C3	1.7719 (14)	C8—H8A	1.02 (2)
S1—C6	1.7929 (16)	C8—H8B	1.05 (2)
O6—Li1^i^	1.969 (3)	C9—C10	1.541 (2)
C3—C4	1.526 (2)	C9—H9A	0.99 (2)
C3—H3A	0.98 (2)	C9—H9B	0.95 (2)
C3—H3B	0.89 (2)	C10—H10A	0.933 (19)
C4—C5	1.536 (2)	C10—H10B	0.92 (2)
			
O7—Li1—O5	100.44 (13)	C6—C5—H5A	109.7 (14)
O7—Li1—O1	138.48 (16)	C4—C5—H5A	106.3 (14)
O5—Li1—O1	107.32 (13)	C6—C5—H5B	110.0 (13)
O7—Li1—O6^i^	103.16 (13)	C4—C5—H5B	114.6 (12)
O5—Li1—O6^i^	103.55 (14)	H5A—C5—H5B	108.8 (18)
O1—Li1—O6^i^	99.66 (13)	C5—C6—S1	105.21 (11)
C1—O1—Li1	129.39 (15)	C5—C6—H6A	110.8 (13)
C1—O2—B1	109.35 (12)	S1—C6—H6A	105.9 (13)
C2—O4—B1	110.03 (12)	C5—C6—H6B	114.9 (12)
O1—C1—O2	126.71 (16)	S1—C6—H6B	106.6 (12)
O1—C1—C2	124.62 (15)	H6A—C6—H6B	112.7 (18)
O2—C1—C2	108.66 (13)	O8—S2—O7	115.66 (7)
O3—C2—O4	126.78 (18)	O8—S2—C7	109.79 (8)
O3—C2—C1	125.12 (16)	O7—S2—C7	111.29 (7)
O4—C2—C1	108.09 (13)	O8—S2—C10	108.96 (7)
F2—B1—F1	111.75 (13)	O7—S2—C10	112.75 (7)
F2—B1—O4	110.44 (13)	C7—S2—C10	96.78 (7)
F1—B1—O4	111.20 (13)	S2—O7—Li1	144.93 (11)
F2—B1—O2	109.78 (13)	C8—C7—S2	102.27 (11)
F1—B1—O2	109.62 (13)	C8—C7—H7A	115.0 (13)
O4—B1—O2	103.76 (12)	S2—C7—H7A	109.7 (13)
O6—S1—O5	116.62 (7)	C8—C7—H7B	112.9 (14)
O6—S1—C3	111.18 (7)	S2—C7—H7B	104.0 (14)
O5—S1—C3	109.29 (8)	H7A—C7—H7B	112.0 (19)
O6—S1—C6	112.38 (7)	C7—C8—C9	106.42 (13)
O5—S1—C6	108.18 (7)	C7—C8—H8A	112.5 (11)
C3—S1—C6	97.48 (8)	C9—C8—H8A	110.6 (11)
S1—O5—Li1	137.86 (11)	C7—C8—H8B	108.6 (13)
S1—O6—Li1^i^	134.30 (10)	C9—C8—H8B	109.5 (13)
C4—C3—S1	102.68 (10)	H8A—C8—H8B	109.1 (17)
C4—C3—H3A	114.1 (13)	C8—C9—C10	109.02 (13)
S1—C3—H3A	107.2 (13)	C8—C9—H9A	107.7 (13)
C4—C3—H3B	116.6 (13)	C10—C9—H9A	109.7 (13)
S1—C3—H3B	106.4 (13)	C8—C9—H9B	111.8 (13)
H3A—C3—H3B	108.9 (18)	C10—C9—H9B	110.2 (13)
C3—C4—C5	105.72 (13)	H9A—C9—H9B	108.4 (18)
C3—C4—H4A	112.4 (12)	C9—C10—S2	105.47 (10)
C5—C4—H4A	107.4 (12)	C9—C10—H10A	113.2 (12)
C3—C4—H4B	110.9 (14)	S2—C10—H10A	108.0 (12)
C5—C4—H4B	113.9 (14)	C9—C10—H10B	115.4 (13)
H4A—C4—H4B	106.6 (18)	S2—C10—H10B	105.9 (13)
C6—C5—C4	107.33 (13)	H10A—C10—H10B	108.4 (18)
			
O7—Li1—O1—C1	24.4 (3)	C3—S1—O6—Li1^i^	−179.61 (15)
O5—Li1—O1—C1	−105.29 (18)	C6—S1—O6—Li1^i^	72.30 (16)
O6^i^—Li1—O1—C1	147.13 (16)	O6—S1—C3—C4	−143.40 (10)
Li1—O1—C1—O2	−169.91 (15)	O5—S1—C3—C4	86.46 (12)
Li1—O1—C1—C2	9.1 (3)	C6—S1—C3—C4	−25.83 (12)
B1—O2—C1—O1	−177.70 (15)	S1—C3—C4—C5	44.85 (14)
B1—O2—C1—C2	3.12 (16)	C3—C4—C5—C6	−47.70 (17)
B1—O4—C2—O3	−179.29 (16)	C4—C5—C6—S1	27.11 (16)
B1—O4—C2—C1	−0.31 (16)	O6—S1—C6—C5	116.13 (11)
O1—C1—C2—O3	−2.0 (3)	O5—S1—C6—C5	−113.69 (12)
O2—C1—C2—O3	177.15 (15)	C3—S1—C6—C5	−0.49 (13)
O1—C1—C2—O4	178.94 (15)	O8—S2—O7—Li1	−128.77 (19)
O2—C1—C2—O4	−1.86 (17)	C7—S2—O7—Li1	105.0 (2)
C2—O4—B1—F2	119.62 (14)	C10—S2—O7—Li1	−2.5 (2)
C2—O4—B1—F1	−115.73 (14)	O5—Li1—O7—S2	52.0 (2)
C2—O4—B1—O2	2.03 (16)	O1—Li1—O7—S2	−79.7 (3)
C1—O2—B1—F2	−121.27 (14)	O6^i^—Li1—O7—S2	158.69 (13)
C1—O2—B1—F1	115.62 (14)	O8—S2—C7—C8	80.33 (12)
C1—O2—B1—O4	−3.23 (16)	O7—S2—C7—C8	−150.31 (10)
O6—S1—O5—Li1	−38.91 (18)	C10—S2—C7—C8	−32.65 (12)
C3—S1—O5—Li1	88.21 (17)	S2—C7—C8—C9	45.93 (16)
C6—S1—O5—Li1	−166.71 (16)	C7—C8—C9—C10	−41.02 (19)
O7—Li1—O5—S1	−172.88 (11)	C8—C9—C10—S2	16.08 (17)
O1—Li1—O5—S1	−24.1 (2)	O8—S2—C10—C9	−103.64 (12)
O6^i^—Li1—O5—S1	80.71 (19)	O7—S2—C10—C9	126.54 (11)
O5—S1—O6—Li1^i^	−53.42 (17)	C7—S2—C10—C9	10.02 (12)

Symmetry codes: (i) −*x*+1, −*y*, −*z*.
